# IgA nephropathy in patients with serum anti-neutrophil cytoplasmic autoantibody (ANCA) positivity: case series

**DOI:** 10.1590/2175-8239-JBN-2021-0018

**Published:** 2021-07-21

**Authors:** Cristiane Bitencourt Dias, Lectícia Barbosa Jorge, Viktoria Woronik, Lívia Barreira Cavalcante, Luis Yu

**Affiliations:** 1 Universidade de São Paulo, Faculdade de Medicina, Hospital das Clínicas, Laboratório de Fisiologia Renal, São Paulo, SP, Brasil.; 2 Universidade de São Paulo, Faculdade de Medicina, Hospital das Clínicas, Serviço de Nefrologia, São Paulo, SP, Brasil.; 3 Universidade de São Paulo, Faculdade de Medicina, Hospital das Clínicas, Departamento de Patologia, São Paulo, SP, Brasil.

**Keywords:** Glomerulonephritis, IGA, Antibodies, Antineutrophil Cytoplasmic, Biopsy, Glomerulonefrite por IGA, Anticorpos Anticitoplasma de Neutrófilos, Biópsia

## Abstract

Some cases of patients with IgA nephropathy diagnosed via kidney biopsy and antineutrophil cytoplasmic antibody (ANCA) positivity have been reported. This article describes a case series comprising patients with IgA nephropathy and ANCA positivity seen at a medical center in the city of São Paulo, Brazil, from 1996 to 2016. A total of 111 patients underwent diagnostic kidney biopsies for IgA nephropathy. Five were ANCA-positive at the time of diagnosis; their mean age was 45 ± 15.3 years and they were predominantly females with a mean proteinuria of 2.2 ± 0.9 g/day and a median serum creatinine level of 2.5 (2.0 - 8,6) mg/dL; all had hematuria. Four of the five were cANCA-positive (80%); all had normal serum C3 and C4 levels; and 80% were positive for ANA. One case presented an association with infection, but no associations were found with medication. One patient had granuloma and another had a collapsing lesion. This article describes the cases of five ANCA-positive patients (with predominantly cANCA positivity) submitted to diagnostic kidney biopsies for IgA nephropathy; one patient had a collapsing lesion, but progressed well.

## Resumo

Alguns casos clínicos de biópsia renal diagnóstica de nefropatia por IgA em pacientes com títulos séricos positivos de anticorpo anticitoplasma de neutrófilos (ANCA) vêm sendo publicados. Descreve-se uma série de casos de nefropatia por IgA com ANCA positivo de centro único da cidade de São Paulo, Brasil, no período de 1996 a 2016. No período estudado, houve 111 pacientes com biópsia renal com diagnóstico de nefropatia por IgA; destes, 5 tinham ANCA positivo ao diagnóstico com média de idade de 45 ± 15,3 anos, predominando o sexo feminino, com média de proteinúria de 2,2 ± 0,9 g/dia, hematúria presente em 100% dos casos e mediana de creatinina sérica de 2,5 (2,0 - 8,6) mg/dL. O cANCA foi o padrão mais encontrado, em 4 dos 5 casos (80%), com os níveis séricos das frações de complemento C3 e C4 normais em todos e FAN positivo em 80% dos casos. Houve associação com infecções em um caso, mas sem associação com medicações. À microscopia óptica, um dos pacientes tinha granuloma e outro, lesão colapsante. Em resumo, descreve-se cinco casos de pacientes com biopsia renal diagnóstica de nefropatia por IgA com ANCA sérico positivo predominando cANCA, destacando um paciente desse grupo com microscopia óptica com lesão colapsante que, apesar disso, teve boa evolução.

## Introduction

IgA nephropathy (IgAN) is the most common type of glomerulopathy. Its pathogenesis has been linked to the formation of anomalous immunoglobulin A (IgA). The reported frequency of occurrence of the association between IgAN and antineutrophil cytoplasmic antibody (ANCA) positivity is 1.2%; this combination has not been linked to poorer outcomes^1^.

The pathophysiology of IgAN in ANCA-positive patients is unclear. Some believe that IgA found in ANCA-positive patients and biopsies with signs of crescentic glomerulonephritis is not pathogenic, and that the actual disease is ANCA-associated vasculitis (AAV). This hypothesis was developed based on postmortem studies in which IgA deposits were found in the kidneys of individuals without a history of kidney disease. Another idea is that ANCA is not pathogenic, since in some kidney biopsies no crescents are found, a necessary condition to establish a diagnosis of AAV. And finally, some have pondered about the existence of an association between the two diseases^2^.

A study carried out in Europe reported a prevalence of 2% for the association of IgAN and ANCA positivity. The authors also described three cases of lung involvement, an unexpected development in cases of IgAN that might place ANCA as the culprit in the genesis of the disease^3^. In the introduction to their article, Hass M et al. discussed the existence of atypical ANCA patterns, with specific reference to pANCA directed against elastase and lactoferrin, found in 80% of the cases of ulcerative colitis (UC), 70% of the patients with sclerosing cholangitis, 40% of the individuals with Crohn's disease, and in subjects with endocarditis. Four of the six cases of IgAN and ANCA positivity described in their study involved cANCA-positive (anti-proteinase 3) patients, two of which had lung involvement, thereby suggesting a role for the antibody in the onset of disease^4^.

Given the relevance of the subject and the absence of publications describing populations in South America, this article was written to describe a series of cases of IgAN and ANCA positivity seen at a medical center in the city of São Paulo, Brazil.

## Material and Methods

This retrospective study included the kidney biopsies of patients diagnosed with IgAN with at least eight glomeruli per sample seen at the Nephrology Department of the Hospital das Clínicas of São Paulo from 1996 to 2016. The data of patients with IgAN and ANCA positivity were processed to extract information such as age, sex, presence of systemic symptoms, hematuria defined as presence of three or more red blood cells per High Power Field, 24-hour urinary protein or urine protein to creatinine ratio, hemoglobin, serum creatinine, glomerular filtration rate (GFR) estimated with the CKD-EPI equation, complement C_3_ and C_4_, ANCA staining pattern using indirect immunofluorescence, antinuclear antibodies (ANA) also using indirect immunofluorescence, and viral serology.

Light microscopy images of kidney biopsy specimens were assessed for the presence of glomerular crescents, mesangial and endocapillary proliferation, degree of tubular atrophy and interstitial fibrosis, in addition to presence or absence of vascular involvement. Immunofluorescence staining images were assessed for intensity and location of immunoglobulins and complement fractions. IgAN was defined as the presence of deposits of immunoglobulin A in a predominant or codominant pattern in the mesangium seen with immunofluorescence staining. The patients were also rated based on the Oxford Classification (MEST-C score) as M0 or M1 (mesangial cellularity in < 50% or ≥ 50% of the glomeruli, respectively); E0 or E1 for absence of presence of endocapillary hypercellularity, respectively; S0 or S1 for absence or presence of segmental glomerulosclerosis, respectively; T0, T1 or T2, for presence of tubular atrophy or interstitial fibrosis in up to 25%, 26%-50%, or in more than 50% of the cortical area, respectively; and C0, C1 or C2 for absence of crescents, crescents in up to 24% of the glomeruli, or crescents in more than 24% of the glomeruli, respectively^5^.

Outcome data included prescribed therapy and tests performed six months after diagnosis and at the end of follow-up, defined as the last visit to the service or the end date of this study. The tests analyzed in these two time periods looked into hematuria, proteinuria, serum creatinine, ANCA, and referral to renal replacement therapy (RRT).

Numerical data were presented as mean values ± standard deviation or median values (interquartile range) when appropriate; categorical data were presented as absolute numbers or proportions. The Nephrology Department of the institution approved the study.

## Results

A total of 111 patients underwent diagnostic kidney biopsies for IgAN, of which five were ANCA-positive (4.5%). Table 1 describes the clinical and workup data for each of these patients; noteworthy findings include patient mean age (45 ± 15.3 years); there was only one patient aged 60+ years; patients were predominantly females (80%); mean 24-hour urinary protein was 2.2 ± 0.9 g/day; all patients had hematuria; and their median serum creatinine was 2.5 (2.0 - 8.6) mg/dL (GFR: 22(6-24) mL/min/1.73m^2)^. The most common staining pattern was cANCA (80%); all had normal serum C_3_ and C_4_ levels; and 80% were positive for ANA (Table 1).

**Table 1:** Clinic and Biochemistre datas of IgA Nephropathy with ANCA positive patients at diagnosis

Patients	Age (years)	Sex (F/M)	GFR (mL/min/1.73m^2^)	Hematuria (erythrocytes/field)	Proteinuria (g/day)	Serum albumine (g/dL)	Hemoglobine (g/dL)	ANCA	C_3_ (mg/dL)	C_4_ (mg/dL)	ANA	HIV	Virus B	Virus C	Systemic manifestation
1	44	F	22	13	2.8	3.2	11	cANCA	98	29,6	pos	neg	neg	neg	RA 11 years ago
2	70	F	24	64	1.2	4.3	10.8	pANCA	125	40,6	pos	neg	neg	pos	viral charge undetectable without treatment
3	41	M	5	86	1.3	2.9	6.2	cANCA	137	36	pos	neg	neg	neg	febre
4	42	F	79	100	2.76	3.5	13.9	cANCA	153	14,7	neg	neg	neg	neg	neg
5	28	F	6	100	3.2	3.6	9.6	cANCA	115	28	pos	neg	neg	neg	osteomielite

Total	45±15,3	80% F	22(6-24)	100% pos	2.2±0.9	3.5 ±0 .5	10.3±2.7	cANCA 80%	125,6 ±2 1	39,7 ± 9,8	Pos 80%	Neg 100%	Neg 100%	Pos 20%	

F- female; M- male; Pos- positive; neg- negative; RA- rheumatoid arthritis. GFR - glomerular filtration rate by CKDEPI. Mean±SD ou median (interquartile range)

In regard to viral serology, all patients were negative for hepatitis B virus and HIV; one patient tested positive for hepatitis C virus, with a negative viral load and without prior treatment. One patient had fever, the only systemic manifestation seen in our cohort; one patient had a history of left femur osteomyelitis by Staphylococcus sp.; one patient had been previously diagnosed with rheumatoid arthritis (Table 1).

Table 2 describes kidney biopsy findings. Three patients had glomerular crescents, although only Patient 3 had light microscopy findings suggestive of AAV, since in addition to having a larger proportion of crescents, the patient did not have other types of proliferation such as mesangial or endocapillary, and presented with fibrinoid necrosis and granuloma. The Oxford Classification scores of the other patients were as follows: M0: 60%; E0: 100%, S1: 80%; and T0: 80%. Patients 1 and 2 did not have crescents, while Patients 3, 4, and 5 had 28,5%, 10%, and 20% involvement, respectively. Patient 1 had one glomerulus with a collapsing lesion characterized by podocyte hyperplasia and hypertrophy and glomerular loop collapse. Immunofluorescence tests showed a staining pattern consistent with IgAN in all patients, with predominant or codominant deposits of IgA in the mesangium and C3 deposits in the mesangium, with the exception of Patients 2 and 5, who had only IgA deposits without C3 (Table 2).

**Table 2:** Characteristics of renal histology using the Oxford classification and immunofluorescence of all patients

Patients	Mesangial proliferation (M)	Endocapillary proliferation (E)	Segmental sclerosis (S)	Tubular Atrophy and Fibrosis (T)	Crescents (C)	Vessel involvement	Other findings	IgG	IgA	IgM	C3	C1q
1	0	0	1	1	0	yes	collapsing lesion	neg	3+ mesangial	1+ mesangial	3+ mesangial	neg
2	1	0	1	0	0	yes		neg	1+ mesângial	neg	neg	neg
3	0	0	1	0	2	yes	fibrinoid necrosis and granuloma	neg	3+ mesangial	neg	1+ mesangial	neg
4	1	0	1	0	1	yes		neg	2+ mesangial	neg	1+ mesangial	neg
5	0	0	0	0	1	yes		neg	1+ mesangial	neg	neg	neg

neg- negative

**Graph 1:**
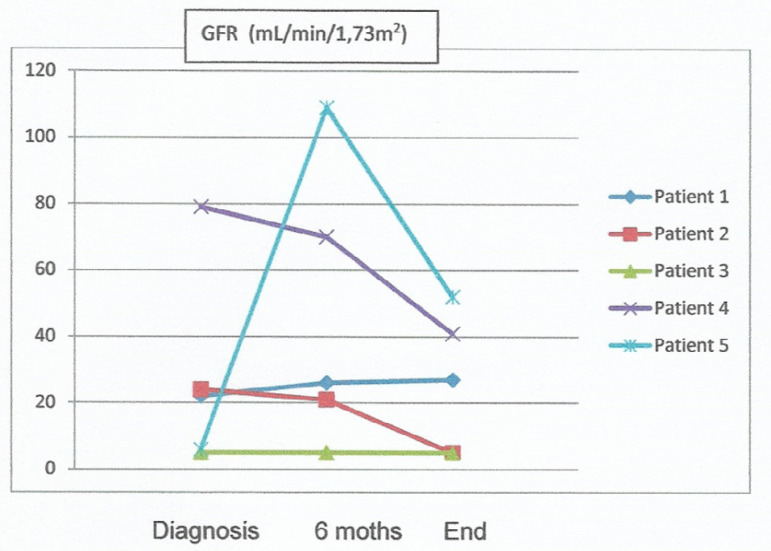
Evolution of creatinine clearence of each patient at diagnosis, at six months and at the end of follow up. GFR Glomerular Filtration rate by CKDEPI.

**Graph 2:**
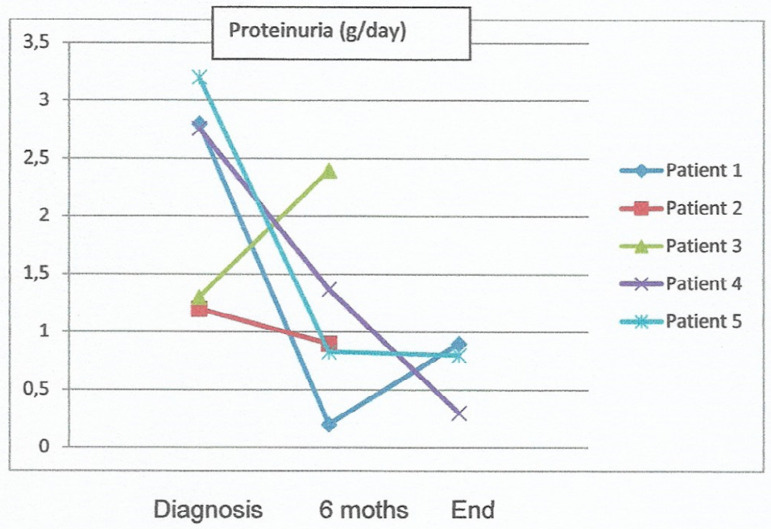
Evolution of proteinuria in each patient at diagnosis, at six months and at the end of follow -up

In regards to treatment, all patients were given renin-angiotensin-aldosterone system inhibitors and Patients 3 and 5 were also prescribed immunosuppressant therapy: steroids and cyclophosphamide to Patient 3 and steroids only to Patient 5. Patient 3 has been on RRT since diagnosis. Six months after diagnosis, Patients 1, 2, 4, and 5 had an average serum creatinine level of 1.5 ± 0.8 mg/dL (GFR 26 (21-70) ml/min/1.73m^2)^; hemoglobin 119 ± 1.2 g/dL; 24-hour urinary protein 1.1 ± 0.8 g/day; only Patient 2 remained ANCA-positive and hematuria ceased only in Patient 1. The patients were followed on for 21 (12 - 60) months on average, with Patient 2 requiring RRT. Creatinine clearance remained stable in Patient 1 and Patient 4 showed declining kidney function without requiring RRT; the two had 24-hour urinary protein levels below 1g/day, did not have hematuria and were ANCA-negative. Kidney function improved in Patient 5, with a serum creatinine level of 1.3 mg/dL and 24-hour urinary protein of 0.8 g/day. Graph 1 shows the progression of creatinine clearance calculated by the CKD-EPI equation for each patient, while Graph 2 shows the progression of 24-hour urinary protein levels.

## Discussion

Infection by Staphylococcus sp., in cases of endocarditis or osteomyelitis involving patients with diabetes and older individuals in particular, have been described as an element in the etiology of glomerulonephritis with predominant IgA staining and ANCA positivity^6^. The presence of IgA deposits in the kidney biopsies of patients with staphylococcal infection has a well defined pathogenesis, while ANCA has been related to significant exposure to neutrophil antigens caused by the massive destruction of neutrophils during infection^6^. The authors usually do not call this condition, when associated with infection, IgA nephropathy, but a post-infection presentation with predominant IgA staining that resolves as the infection is treated^6^. In our study, Patient 5 had an associated infection that nonetheless evolved well, as the patient moved from a creatinine clearance of 6 mL/min/1.73m^2^ to 52 mL/min/1.73m^2^ at the end of follow-up, which in the case of this patient was 60 months.

In a study including patients with IgA vasculitis, the so-called Henoch-Schönlein purpura, ANCA positivity - pANCA only - was seen in 5.8% of the patients, affecting older patients with more significant pulmonary and neurological involvement^7^. It has been posited that ANCA is the outcome of a cytokine storm that might affect some patients, leading to the release of "antigens" recognized by ANCA. Since pulmonary and neurological involvement are uncommon in IgA vasculitis, the authors assumed ANCA might have a role in the pathogenesis of this condition^7^. The patients included in our study did not meet the criteria for IgA vasculitis.

Some authors compared series comprising patients with IgAN and ANCA positivity to series involving individuals with IgAN and negative ANCA tests, each using some form of randomization. None showed worse progression in terms of kidney survival associated with ANCA positivity^1^^,^ 3 ^,^ 8. The study by Xie L et al. compared 35 cases of IgAN with ANCA positivity to 40 randomly picked patients with IgAN and negative ANCA tests, and found that kidney survival outcomes were not unfavorable to individuals with ANCA positivity. The study in question also further segregated patients with IgAN and ANCA positivity with systemic manifestations (n=14) from individuals without systemic manifestations (n=21), and found no difference in terms of renal outcomes^8^.

Studies looking exclusively into cases of AAV differentiate between antimyeloperoxidase-associated vasculitis, usually pANCA-positive, and antiproteinase 3-associated vasculitis, a cANCA-positive condition. The rationale behind confronting indirect immunofluorescence data providing cytoplasmic or perinuclear ANCA staining patterns and ELISA results defining the antigen is based, for pANCA in particular, on the fact that antigens other than antimyeloperoxidase might be present^4^.

Medication is often described as a cause of autoimmune disease. Hydralazine, a drug used to treat high blood pressure, has been implicated in cases of lupus and AAV, although it has not been associated with IgAN. Hydralazine would inhibit DNA methylation and thereby increase the expression of neutrophil antigens^9^. Our patients were not prescribed medications such as hydralazine or alopurinol, and they did not have a history of taking illicit drugs prior to diagnosis.

In our series, only Patient 3 appeared to have a pathogenic ANCA, since in addition to crescents (epithelial proliferation) seen in light microscopy the subject also had fibrinoid necrosis, granuloma, and absence of mesangial or endocapillary proliferation typically seen in individuals with IgAN. The kidney biopsy findings of the remaining patients were consistent with IgAN, with mesangial proliferation even in M0 patients according to the Oxford Classification^5^; two patients did not have crescents.

Patient 1 had IgAN with collapsing lesions and cANCA positivity, a rare occurrence described in the literature only in association with the use of anabolic steroids and collapsing glomerulopathy with predominant IgA staining, although not associated with ANCA^10^.

There is a rare description of IgAN and AAV in patients exposed to silica^11^, which pathogenesis is still discussed. None of our patients had any type of exposure prior to diagnosis. On the renal outcomes of these patients, studies comparing ANCA-positive IgAN to AAV or IgAN have not reported differences in kidney survival^2^^,^ 12 ^)^ .

This paper described the cases of five patients diagnosed with IgAN based on kidney biopsies and the identification of predominant or codominant staining patterns of immunoglobulin A deposits in the mesangium, with ANCA positivity - predominantly cANCA; light microscopy findings of one patient revealed signs of collapsing lesions, which eventually progressed to a good outcome.
